# Matrix metalloproteinase 11 protects from diabesity and promotes metabolic switch

**DOI:** 10.1038/srep25140

**Published:** 2016-04-29

**Authors:** Nassim Dali-Youcef, Karim Hnia, Sébastien Blaise, Nadia Messaddeq, Stéphane Blanc, Catherine Postic, Philippe Valet, Catherine Tomasetto, Marie-Christine Rio

**Affiliations:** 1 Department of Functional Genomics and Cancer, Institut de Génétique et Biologie Moléculaire et Cellulaire (IGBMC)/ CNRS UMR 7104/ INSERM U 964/ Université de Strasbourg, 1 rue Laurent Fries, 67404 Illkirch, France; 2 Laboratoire de Biochimie et Biologie Moléculaire, Pôle de Biologie, Hôpitaux Universitaires de Strasbourg, 1 place de l’hôpital, 67098 Strasbourg Cedex, France; 3 Department of Translational Medicine and Neurogenetics, Institut de Génétique et Biologie Moléculaire et Cellulaire (IGBMC)/ CNRS UMR 7104/ INSERM U 964/ Université de Strasbourg, 1 rue Laurent Fries, 67404 Illkirch, France; 4 Institut des Maladies Métaboliques et Cardiovasculaires, INSERM U1048, Université de Toulouse, UPS, Toulouse, France; 5 Electron microscopy platform, Institut de Génétique et Biologie Moléculaire et Cellulaire (IGBMC)/ CNRS UMR 7104/ INSERM U 964/ Université de Strasbourg, 1 rue Laurent Fries, 67404 Illkirch, France; 6 Institut Pluridisciplinaire Hubert Curien, UMR 7178, CNRS-IN2P3-INC-INEE, Université de Strasbourg, 23 rue du Loess-BP28, 67037 Strasbourg cedex 2, France; 7 INSERM, U1016, Institut Cochin/ CNRS, UMR8104/ Université Paris Descartes, Sorbonne Paris Cité, 75014, Paris, France

## Abstract

MMP11 overexpression is a bad prognostic factor in various human carcinomas. Interestingly, this proteinase is not expressed in malignant cells themselves but is secreted by adjacent non-malignant mesenchymal/stromal cells, such as cancer associated fibroblasts (CAFs) and adipocytes (CAAs), which favors cancer cell survival and progression. As MMP11 negatively regulates adipogenesis *in vitro*, we hypothesized that it may play a role in whole body metabolism and energy homeostasis. We used an *in vivo* gain- (Mmp11-Tg mice) and loss- (Mmp11−/− mice) of-function approach to address the systemic function of MMP11. Strikingly, MMP11 overexpression protects against type 2 diabetes while Mmp11−/− mice exhibit hallmarks of metabolic syndrome. Moreover, Mmp11-Tg mice were protected from diet-induced obesity and display mitochondrial dysfunction, due to oxidative stress, and metabolic switch from oxidative phosphorylation to aerobic glycolysis. This Warburg-like effect observed in adipose tissues might provide a rationale for the deleterious impact of CAA-secreted MMP11, favouring tumor progression. MMP11 overexpression also leads to increased circulating IGF1 levels and the activation of the IGF1/AKT/FOXO1 cascade, an important metabolic signalling pathway. Our data reveal a major role for MMP11 in controlling energy metabolism, and provide new clues for understanding the relationship between metabolism, cancer progression and patient outcome.

More and more epidemiological data attribute a pernicious effect to metabolic disorders, notably obesity and diabetes, on cancer progression. Moreover, metformin, a drug commonly administered for type 2 diabetes, displays antitumor properties^1^. To date, most studies have emphasized tumor aggressiveness in a context of already constituted metabolic disorders. Less attention has been given to the ability of cancer cells to impact on metabolism via molecules abnormally expressed by primary tumors and/or metastases. This effect may be of interest and should therefore be evaluated to unravel the link between metabolism and cancer.

Matrix metalloproteinase 11 (MMP11; previously named stromelysin-3)^2^ belongs to a family of extracellular proteolytic enzymes involved in extracellular matrix (ECM) remodeling and activation of latent factors. While most MMPs require the presence of additive proteinases to be extracellularly activated, MMP11 is activated prior to secretion by Golgi-associated furin-like proteinases^3^,^4^. From a functional point of view, MMP11 is a unique member of the MMP family. Indeed, it is a very selective enzyme that does not cleave major components of the ECM. To date, only three main substrates have been identified, namely the insulin-like growth factor-binding protein-1 (IGFBP-1)^5^, the laminin receptor^6^ and the native alpha3 chain of collagen VI^7^. This sharp specificity is probably due to its particular S1′ pocket structure looking like a tunnel running through the enzyme^8^.

MMP11 plays a role in various embryonic and adult physiological processes, including postnatal mammary gland development and function^9^. MMP11 also participates in human diseases, most notably cancers. MMP11 overexpression occurs in various human invasive carcinomas including the common breast, prostate and colon cancers. High MMP11 levels correlate with tumor aggressiveness as well as with poor patient clinical outcome^10^,^11^. Importantly, MMP11 is a tumor proteinase not expressed in the malignant epithelial cells, but in the non-malignant adjacent mesenchymal/stromal cells^12^. Chemically-induced^12^ or ras oncogene-induced^13^ mouse tumor models have shown that stromal MMP11 is a key factor for tumor progression. It lowers cancer cell death through anoikis during local invasion, favouring thereby cancer cell survival and implantation in connective tissues^14^,^15^,^16^. More recently, MMP11 was shown to be expressed by a newly defined cellular component of the tumor microenvironment, called the cancer-associated adipocytes (CAAs), in human cancers^9^,^16^,^17^. Moreover, *in vitro*, MMP11 reduces adipocyte differentiation and even reverts mature adipocyte differentiation^16^. Thus, invasive cancer cells induce proximal adipocytes to express MMP11 leading to their dedifferentiation into fibroblasts [reviewed in^18^]. Tumor resident adipocytes are therefore local regulators of cancer cell behavior via an « adipocyte-cancer cell paracrine loop (ACCPL) » occurring during the tumor invasive steps^19^. The molecular mechanisms involved remain largely unknown.

In this context, we hypothesized that the high amounts of MMP11 present in almost all invasive tumors might impact patient metabolism and energy homeostasis. We have addressed this question in the present study using a genetic strategy. Experiments have been done in the absence of malignancy to discard any possible tumor-related interfering effects. To do so, we developed a MMP11-overexpressing transgenic mouse strain (Mmp11-Tg). We also used MMP11 deficient mice (Mmp11−/−) previously developed in our laboratory^12^. These gain- and loss-of-function models allowed us to examine the *in vivo* function of MMP11 in metabolism. Our data reveal that MMP11 impacts on lipid synthesis and *in vivo* storage in adipose tissues and liver, and protects Mmp11-Tg mice from diabesity. Highly supporting this MMP11 metabolic function, Mmp11−/− mice^12^ displayed a reverse phenotype with hallmarks of type 2 diabetes, most notably glucose intolerance and insulin resistance.

## Results

### Mmp11-Tg mice display decreased fat mass and improved metabolic parameters

**Figure 1:**
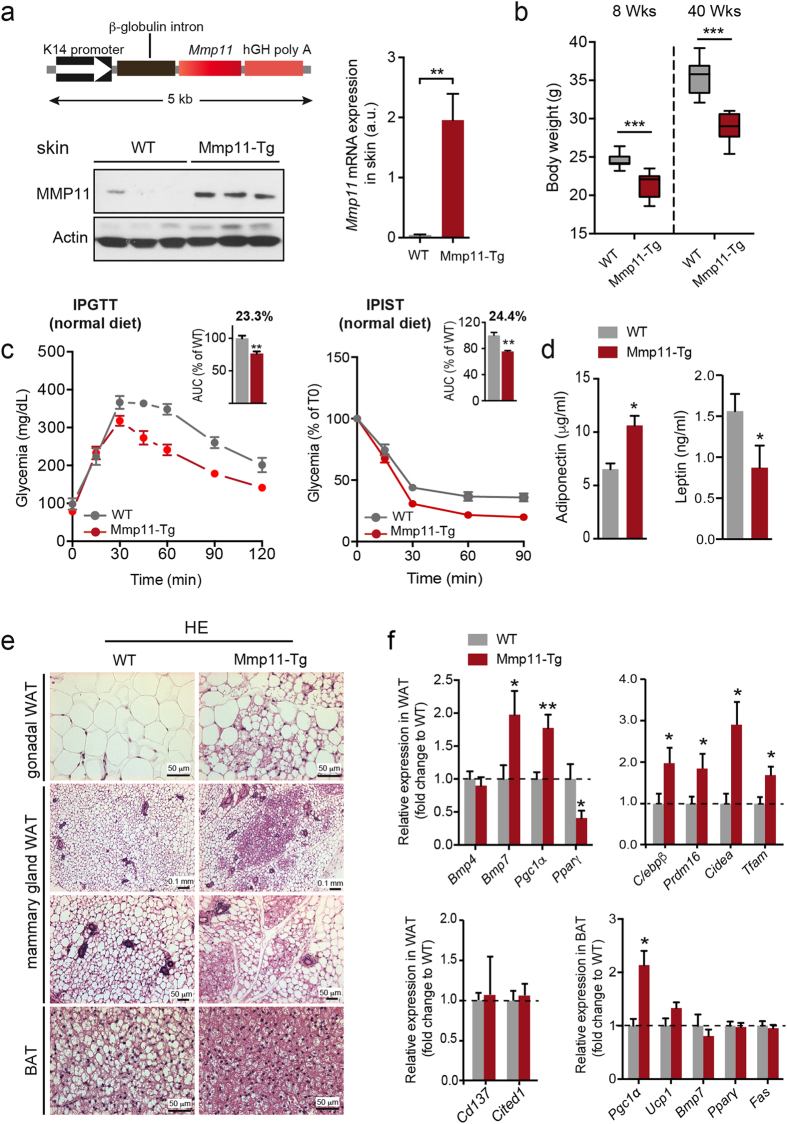
Mmp11-Tg mice metabolic analyses. (**a**) Upper left panel, scheme of the transgene construct. Lower left panel, Western blot showing MMP11 expression in skin protein extracts from 12-week-old WT and Mmp11-Tg mice (n = 3/group); right panel, qRT-PCR analysis showing the robust increase in *Mmp11* mRNA expression in Mmp11-Tg mice compared to controls (n = 5/group); (**b**) Body weight progression in 8- and 40-week-old Mmp11-Tg and WT mice (n = 9/group); (**c**) IPGTT and IPIST analyses and AUC in Mmp11-Tg and WT mice under normal diet conditions; (**d**) Circulating adiponectin and leptin levels in Mmp11-Tg and WT mice (n = 8/group); (**e**) HE staining of gonadal WAT, mammary gland WAT and BAT histological sections from Mmp11-Tg and WT mice. The WAT of Mmp11-Tg mice displayed areas of multilocular brown adipocyte-like cells compared to WT counterparts. The BAT of Mmp11-Tg mice accumulates smaller and less lipid droplets compared to WT animals; (**f**) Expression profile of genes involved in adipogenesis (n = 6–7/group).

We generated transgenic mice, which overexpressed a secreted form of mouse MMP11 (Mmp11-Tg, FVBN genetic background) in the skin, a highly vascularized organ (Fig. 1a, upper left panel). MMP11 protein was detected in skin homogenates from Mmp11-Tg mice, while no expression was detected in controls (Fig. 1a, lower left panel). Robust *Mmp11* mRNA expression was confirmed as well (Fig. 1a, right panel). The level of expression was similar to that detected in human invasive tumors and/or metastases^2^,^10^. Interestingly, body weight was significantly decreased in young (8-week-old) and old (40-week-old) Mmp11-Tg mice compared to WT littermates (Fig. 1b), and the difference more pronounced in older mice. Metabolic profiling indicated that 12-week-old Mmp11-Tg mice had decreased levels of serum glucose, total cholesterol (mainly HDL-cholesterol), triglycerides and FFA (Fig. S1). Consistent with decreased circulating glucose levels, Mmp11-Tg mice exhibited enhanced glucose tolerance and insulin sensitivity compared to WT mice under normal diet conditions (Fig. 1c). Additionally, Mmp11-Tg mice displayed increased serum adiponectin levels and decreased leptin levels, consistent with the loss of body weight observed in mutant mice (Fig. 1d). Histological examination of the peri-gonadal white adipose tissue (WAT) of Mmp11-Tg mice revealed aggregates of multilocular brown adipocyte-like cells, while the brown adipose tissue (BAT) exhibited less fat accumulation than that of control mice (Fig. 1e). Consistently, increased expression of the metabolic regulator and late BAT marker, peroxisome proliferator-activated receptor gamma (PPARγ)-coactivator 1α (*Pgc1α*)^20^, was observed (Fig. 1f). Increased expression of bone morphogenetic protein 7 (*Bmp7*), a protein that triggers commitment of mesenchymal progenitor cells to a brown adipocyte lineage^21^, but not *Bmp4*, which induces the commitment of mesenchymal cells into white preadipocytes^22^, was also noted (Fig. 1f). It is worth noting that the brown-like cells present in the WAT of Mmp11-Tg mice were not of “beige” origin since we did not observe any change in the expression of “beige” markers *Cd137* and *Cited 1*^23^,^24^, while they overexpressed BAT determining genes such as *C/ebp*β, *Prdm16* and *Cidea*^23^,^25^,^26^ (Fig. 1f). In addition, expression of the mitochondria DNA replication gene mitochondrial transcription factor A (*Tfam*) was also enhanced in Mmp11-Tg WAT compared to controls. Interestingly, these brown fat-like cells were also observed in the mammary gland adipose tissue of Mmp11-Tg mice (Fig. 1e). Furthermore, we observed decreased mRNA expression of the master adipose tissue differentiating factor *Pparγ* in the WAT but not in the BAT, and enhanced expression of *Pgc1α* in both tissues (WAT and BAT) without significant changes in the expression of its target gene uncoupling protein 1 (*Ucp1*) in the BAT (Fig. 1f). Thus, MMP11 triggers different molecular changes in the WAT and BAT.

These results indicated that MMP11 impacts whole body metabolism and favours fat browning.

### Mmp11-Tg mice exhibit defective adaptive thermogenesis due to altered mitochondrial function

**Figure 2:**
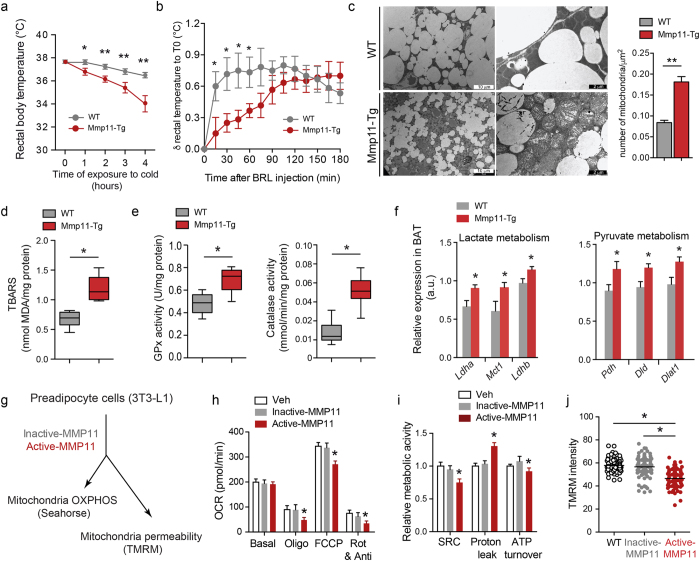
*In vivo* and *in vitro* impact of MMP11 on mitochondrial function. (**a**) Rectal body temperature progression in Mmp11-Tg and WT mice placed during 4 h at 4 °C (n = 5/group); (**b**) Variation in rectal body temperature as a function of time after injection of the β3-adrenergic agonist BRL37344 in Mmp11-Tg and WT mice (n = 6/group); (**c**) TEM showed increased numbers of mitochondria and less fat in the BAT of Mmp11-Tg compared to WT mice. Many of the mitochondria displayed altered cristae (scale bar = 10 μm in left panels and 2 μm in right panels); the mitochondria count in the BAT is indicated (n = 5); (**d**) TBARS measurement in BAT of Mmp11-Tg and WT mice; (**e**) Glutathion peroxidase (GPx) and catalase enzymatic activities in the BAT of Mmp11-Tg and WT mice; (**f**) mRNA expression of genes involved in lactate metabolism and pyruvate metabolism (n = 6–8/group); (**g**) Study of mitochondria function in 3T3-L1 adipocytes treated 24 h with vehicle, active-MMP11 or inactive-MMP11 (OXPHOS = oxidative phosphorylation using Seahorse technology, TMRM = tetramethyl rhodamine methylester for mitochondrial permeability test); (**h**) Oxygen consumption rate (OCR) measurement at baseline and after sequential injection of oligomycin (ATP synthase inhibitor), FCCP (uncoupler agent), and Rotenone/antimycin A (complex I/complex III inhibitors, respectively) in vehicle-, active-MMP11- and inactive-MMP11-treated 3T3-L1 preadipocytes; (**i**) Relative mitochondrial activity parameters (spare respiratory capacity, proton leak and ATP turnover) in vehicle-, active-MMP11- and inactive-MMP11-treated 3T3-L1 preadipocytes; (**j**) Mitochondrial permeability test as measured by TMRM fluorescence intensity, which inversely correlates with the mitochondrial depolarization.

As increases in BAT-like cells could affect thermogenesis, we addressed the ability of Mmp11-Tg mice to maintain a normal body temperature when exposed to cold. After a 4h exposure to cold, Mmp11-Tg mice displayed an inability to maintain body temperature within a physiological range (Fig. 2a). Similarly, an injection of the β3-adrenergic agonist BRL37344 showed an immediate increase in body temperature in WT animals while Mmp11-Tg animals presented a delayed response (Fig. 2b). Despite the presence of BAT-like cells within the WAT, Mmp11-Tg mice have altered adaptive thermogenesis showing that MMP11-induced BAT-like cells were not functional. As mitochondria are the most important supplier of ATP and the site of adaptive thermogenesis, we checked mitochondria structure and function in Mmp11-Tg mice. Ultrastructural analysis by transmission electron microscopy (TEM) showed the presence of proliferating mitochondria displaying altered cristae in BAT cells (Fig. 2c). Based on these observations, we hypothesized that impaired mitochondrial structure and function could be due to enhanced oxidative stress in the BAT of Mmp11-Tg mice. Indeed, we found a significant increase in thiobarbituric acid reactive substances (TBARS), a marker of lipid peroxidation, in the Mmp11-Tg BAT in comparison to controls (Fig. 2d). We then asked whether this oxidative stress was accompanied by an antioxidant defense mechanism. Indeed, we observed an increase in the enzymatic activities of glutathione peroxidase (GPx) and catalase (Fig. 2e), indicating a response to increased oxidative stress.

Since Mmp11-Tg mice had decreased circulating glucose levels and exhibited altered mitochondria, we investigated whether forced MMP11 expression could trigger aerobic glycolysis (i.e. a Warburg-like effect) in the BAT. Indeed, we found that the expression of genes encoding enzymes implicated in lactate production, like LDHA (Lactate Dehydrogenase A) and lactate uptake MCT1 (Monocarboxylate transporter 1), were increased in Mmp11-Tg mice (Fig. 2f). A significant upregulation of LDHB was further noted, suggesting that the lactate produced was most likely converted to pyruvate and used used as an energy fuel. The enhanced expression level of the pyruvate dehydrogenase (*Pdh*) complex, composed of *Dld* (dihydrolipoamide dehydrogenase), *Pdh*, and *Dlat1* (dihydrolipoamide S-acetyltransferase) argues in favour of pyruvate transformation into acetyl coenzyme A (Fig. 2f).

Altogether, these results showed that MMP11 overexpression provoked mitochondrial defects in the BAT, which translated into thermogenesis defects and enhanced expression of glycolytic genes.

### Recombinant MMP11 impairs mitochondrial activity and OXPHOS in 3T3-L1 preadipocytes

Because of the altered mitochondria observed in the BAT of Mmp11-Tg mice, we next addressed the direct effect of MMP11 on mitochondrial function *in vitro*. 3T3-L1 preadipocyte cells were treated for 24 h with active or inactive MMP11 recombinant proteins and key parameters estimating the functionality of oxidative phosphorylation (OXPHOS) were measured (Fig. 2g). The oxygen consumption rate (OCR) was measured at baseline and after sequential injections of oligomycin (an ATP synthase inhibitor), the uncoupling agent FCCP (carbonylcyanide-4-trifluoromethoxyphenylhydrazone) and rotenone/antimycin A (complex I and III inhibitors, respectively). Cells treated with active MMP11 exhibited a significantly lower OCR after oligomycin treatment (Fig. 2h). FCCP treatment also yielded significantly lower maximal respiratory activity compared to cells treated with vehicle or inactive MMP11 (Fig. 2h). As a result, a significant decrease in the spare respiratory capacity (SRC) was observed in active MMP11-treated cells compared to vehicle- or inactive MMP11-treated cells (Fig. 2i). These data show that MMP11 treatment impairs the cellular response to increased energy demand. MMP11 significantly decreased non-mitochondrial respiration (an important parameter for accurate mitochondrial respiration measurement) after injection of rotenone/antimycin A (Fig. 2h) together with a significant increase in proton leak (Fig. 2i), suggesting mitochondrial damage. Moreover, ATP turnover, which represents the rate of oxygen consumption due to mitochondrial ATP synthesis, was significantly decreased in active MMP11-treated cells (Fig. 2i). Consistent with the increase in mitochondrial proton leak, we observed a decrease in the intensity of the mitochondrial fluorescent dye tetramethylrhodamine methyl ester (TMRM) in active MMP11-treated cells indicating an enhanced mitochondria permeability caused by membrane depolarization, a hallmark of metabolically stressed cells (Fig. 2j). Altogether, these *in vitro* data showed that MMP11 impacts negatively on mitochondrial respiratory activity and ATP production.

Our data thus demonstrate that MMP11 negatively affects mitochondrial function through enhanced oxidative stress *in vitro* and *in vivo,* which results in a decrease in mitochondrial respiration and an increase in aerobic glycolysis.

### MMP11 protects from diet-induced obesity (DIO)

**Figure 3:**
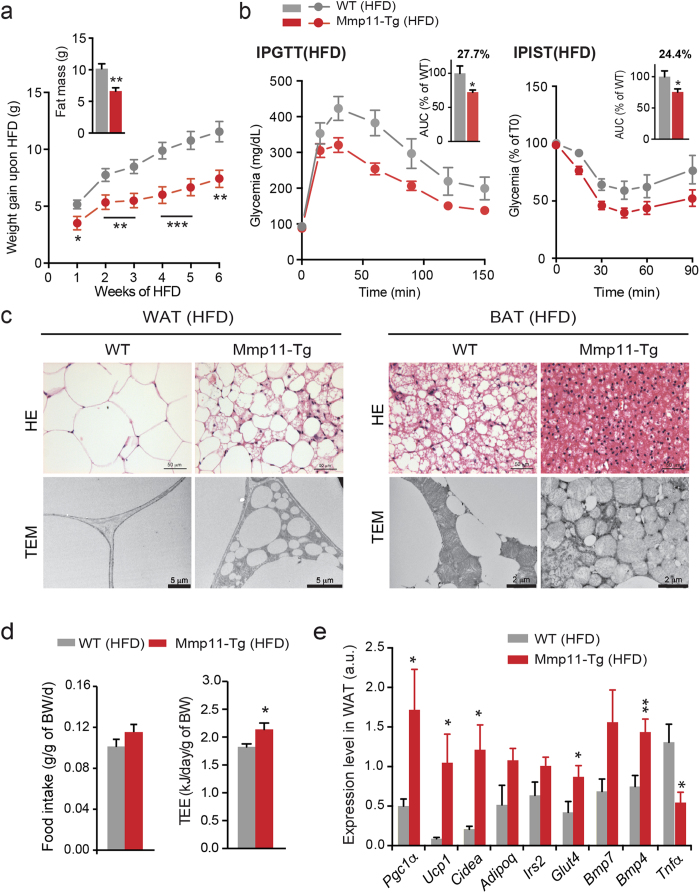
Mmp11-Tg mice are resistant to HFD-induced metabolic syndrome. (**a**) Weight gain progression during 6 weeks of a HFD (n = 13/group) and fat mass measurement at 6 weeks (n = 8–9/group); (**b**) IPGTT (left panel) and IPIST (right panel) tests and AUC (n = 8–10/group) after 6 weeks of a HFD; (**c**) HE and TEM analyses of the WAT (left panel) and BAT (right panel) in Mmp11-Tg and WT mice under HFD; (**d**) Mean daily food intake over 4 days (n = 8–9/group) and total energy expenditure (TEE) quantification at week 5 of the diet (n = 8/group); (**e**) Metabolic gene expression profile in the WAT (n = 8/group).

Having established that MMP11 overexpression modulates fat mass, we aimed to determine whether MMP11 overexpression could prevent the pathophysiological impact of a high fat diet (HFD). Six-week-old Mmp11-Tg mice and control littermates were subjected to a HFD for six weeks. During this time, despite similar food intake (Fig. 3d, left panel), Mmp11-Tg mice were protected from excessive weight gain compared with control mice (Fig. 3a). In addition, under HFD conditions the weight gain differences between Mmp11-Tg and WT mice were more pronounced than under normal diet (ND) conditions. After 6 weeks of HFD, Mmp11-Tg mice exhibited less fat mass than control mice (Fig. 3a, upper panel) and an increase in total energy expenditure (TEE) (Fig. 3d, right panel). Mmp11-Tg mice had improved glucose tolerance and insulin sensitivity, as shown by a smaller area under the curve (AUC) in the intra-peritoneal glucose tolerance (IPGTT) and insulin sensitivity tests (IPIST) (Fig. 3b, left and right panels, respectively). Histological analysis of WAT sections from HFD Mmp11-Tg mice revealed smaller white adipocytes and areas of multilocular brown-like adipocytes compared to the hypertrophied adipocytes of HFD control mice (Fig. 3c, left upper panel). These observations were confirmed by TEM analysis (Fig. 3c, left lower panel). Similarly, the BAT from Mmp11-Tg mice contained very small lipid droplets and was highly enriched in mitochondria, some of which appeared altered (Fig. 3c, right panel).

We then checked the mRNA expression of genes implicated in the adipogenic program in the WAT of HFD-fed Mmp11-Tg mice. We observed increased expression of *Bmp4* recently shown to promote WAT brown fat formation^27^ and some increase for the brown adipocyte marker *Bmp7*^22^ (Fig. 3e). Expression levels of the brown adipocyte markers *Pgc1α*, *Ucp1* and *Cidea* were also enhanced supporting that indeed MMP11 favours WAT browning. Interestingly, a decrease in the expression of *Tnfα* was observed, suggesting a protection against metabolic inflammation in the WAT of Mmp11-Tg mice (Fig. 3e). Further, an increase in the expression of the glucose transporter *Glut4*, *Irs2* and adiponectin (*Adipoq*) was observed in the WAT of Mmp11-Tg mice, in accordance with improved insulin sensitivity (Fig. 3e). Consistently, the livers of Mmp11-Tg mice were also protected from hepatic steatosis compared to WT livers (Fig. S2a upper panel). TEM analysis showed decreased number and size of liver lipid droplets in HFD-fed Mmp11-Tg mice (Fig. S2a lower panel). We observed an enhanced expression of β-oxidation genes such as *Pparα* and its target gene acyl coenzyme A oxidase (*Aco*) (Fig. S2b). Moreover, the expression levels of *Pgc1α* and of *Foxo1* (forkhead transcription box O1, a transcriptional activator of *PPARα*) were increased. The expression levels of two known lipogenic genes, sterol response element binding protein 1c (*Srebp1c*) and carbohydrate response element binding protein (*Chrebp*), were also elevated (Fig. S2b). Increased expression of the fatty acid synthesis genes acetyl-Coenzyme A carboxylase 1 and 2 (*Acc1* and *Acc2*) was also noted, indicating an increased turnover of fatty acids in the Mmp11-Tg liver under HFD. Furthermore, HFD Mmp11-Tg liver displayed higher insulin sensitivity as demonstrated by an enhanced expression of *Irs2*.

In summary, MMP11 protects from excessive weight gain and hepatic steatosis through increased lipid mobilisation and metabolism from fat and liver.

### MMP11 triggers IGF1 signalling via the IGF1/AKT/FOXO1 cascade in adipose tissue

**Figure 4:**
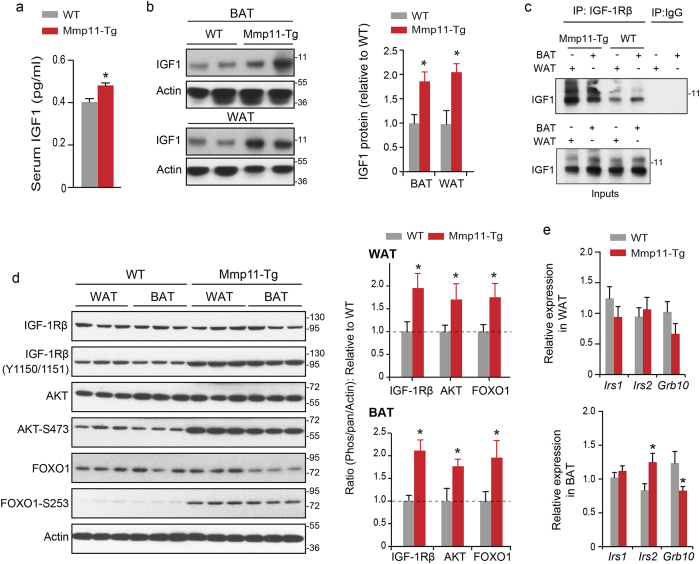
MMP11 activates the IGF1/AKT/FOXO1 axis. (**a**) Circulating IGF1 levels in Mmp11-Tg mice and controls (n = 8/group); (**b**) Immunoblots describing the expression of IGF1 in the WAT and BAT of Mmp11-Tg mice and controls; (**c**) Increased physical interaction between IGF1 and its receptor IGF1Rβ in the WAT and BAT of Mmp11-Tg compared to WT mice. Protein extracts were immunoprecipitated with an IGF1Rβ antibody and the interaction was revealed with an anti-IGF1 antibody; (**d**) left panel: Immunoblots for proteins involved in the IGF1 signalling pathway in the WAT and BAT of Mmp11-Tg mice compared to WT, right panel: quantification of the ratios of phosphorylated IGF1Rβ, pAKT, and pFOXO1 proteins relative to IGF1Rβ, AKT, and FOXO1, respectively, normalized to actin expression (data are presented as fold change of Mmp11-Tg values to WT values); (**e**) Expression profile of genes involved in the insulin/IGF1 pathway in the WAT and BAT of Mmp11-Tg and WT mice (n = 6–7/group).

To uncover the molecular mechanism(s) underlying MMP11 systemic function, we investigated the insulin/IGF1 axis, reported to be involved in metabolic disorders^28^. We observed a significant increase of about 20% in serum IGF1 levels in Mmp11-Tg mice compared to WT animals (Fig. 4a), as well as increases in IGF1 protein levels in WAT and BAT extracts (Fig. 4b). In addition, increased interaction between IGF1 and its receptor IGF1Rβ was found in both WAT and BAT protein extracts (Fig. 4c). Consistently, increased tyrosine phosphorylation of the IGF1Rβ receptor (Y1150/1151) and serine phosphorylation of AKT (S473) indicated that the IGF1/IGF1Rβ pathway was more active in the WAT and BAT of Mmp11-Tg compared to WT (Fig. 4d). As a consequence, FOXO1 phosphorylation (S253), a target of AKT, was also significantly enhanced (Fig. 4d). The level of AKT and FOXO1 were modestly increased in the WAT but not in the BAT of Mmp11-Tg compared to WT mice (Fig. 4d and Fig. S3). Altogether, the overexpression of MMP11 enhances IGF1 bioavailability, which translates into activation of the IGF1/AKT cascade.

In addition to the activation of the IGF1/AKT/FOXO1 signalling pathway, MMP11 overexpression affected the expression of some genes of this pathway, albeit in the BAT but not in the WAT. Indeed, we observed an increase in *Irs2* and a decrease in *Grb10*, a negative regulator of the insulin/IGF1 receptor pathway^29^, mRNA levels in the BAT (Fig. 4e). These observations are in line with an activation of the IGF1/AKT/FOXO1 cascade in Mmp11-Tg mice.

From these experiments, we conclude that MMP11 significantly increases IGF1 bioavailability to affect whole body metabolism.

### Loss of MMP11 induces a metabolic syndrome

**Figure 5:**
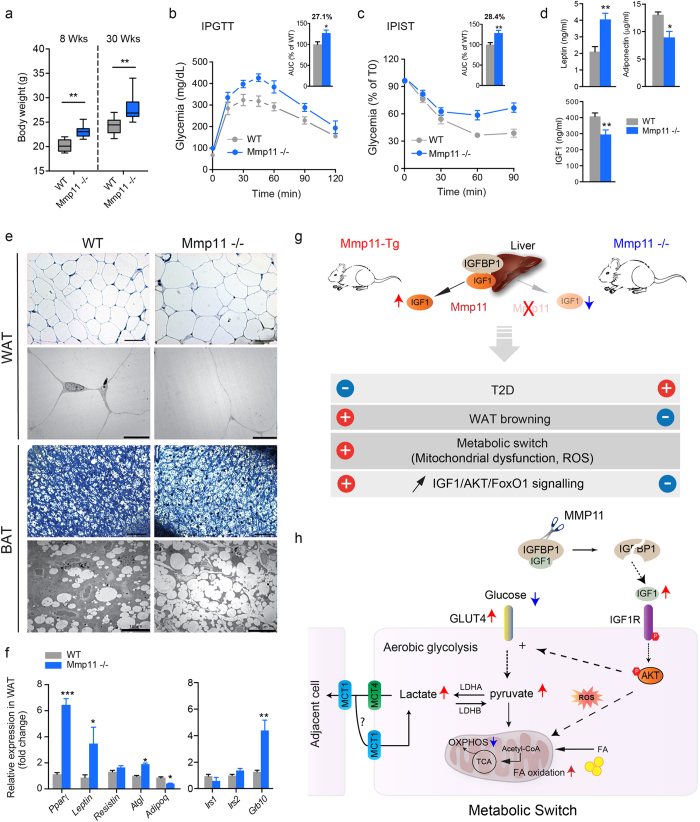
MMP11 deficiency promotes diabesity. (**a**) Increased body weight in 8- and 30-week-old Mmp11−/− mice compared to age-matched WT counterparts (n = 10/group); (**b**) IPGTT test in Mmp11−/− and WT mice (n = 8/group); (**c**) IPIST test in Mmp11−/− and WT mice (n = 8/group); (**d**) Circulating adiponectin, leptin and IGF1 levels in Mmp11−/−and WT mice (n = 8/group); (**e**) Toluidine blue staining (upper panels) and ultrastructure (lower panels) of WAT and BAT. Semi-thin sections (scale bar = 50 μm) and TEM (scale bar = 10 μm) showed hypertrophied white adipocytes in Mmp11−/− mice compared to WT mice. Mmp11−/− brown adipocytes accumulate more fat than WT cells; (**f**) Metabolic gene expression profile changes in the WAT of Mmp11−/− and WT mice (n = 5–7/group); (**g**) Summary of the metabolic abnormalities observed in Mmp11-Tg and Mmp11−/− mice; (**h**) A proposed model of the MMP11-induced metabolic switch from mitochondrial respiration to aerobic glycolysis. MMP11 induces an increase in IGF1 bioavailability. The subsequent increase in the IGF1/AKT signalling provokes an increase in oxidative stress (ROS) which impairs mitochondrial respiration (OXPHOS), and an increase in aerobic glycolysis producing lactate that can be used as a complementary source of energy for the cell itself or for neighboring cells via the MCT4/MCT1 shuttle. Fatty acid oxidation is also increased and represents a metabolic fuel as well. TCA: tricarboxylic acid cycle.

Given that MMP11 overexpression was associated with major metabolic changes, we asked if MMP11 deletion could mirror Mmp11-Tg phenotype. Under normal dietary conditions, we observed that young (8-week-old) and older (30-week-old) MMP11 knockout (Mmp11−/−) mice exhibited significantly increased body weight (Fig. 5a) compared to age-matched wild type mice (WT). With complete penetrance, 12-week-old Mmp11−/−mice displayed significantly increased serum glucose, insulin, total cholesterol, triglycerides (TG) and, FFA levels (Fig. S4a), together with hyperleptinemia and hypoadiponectinemia (Fig. 5d), suggestive of a metabolic syndrome phenotype. The levels of circulating IGF1 were also decreased in Mmp11−/−mice (Fig. 5d). Supporting these data, Mmp11−/−mice showed decreased glucose tolerance (Fig. 5b) and insulin sensitivity (Fig. 5c) compared to control mice. Semi thin sections of Mmp11−/−mice showed hypertrophied adipocytes in the WAT and increased fat depots in the BAT as compared to WT animals (Fig. 5e, upper panels). TEM analysis confirmed that Mmp11−/−BAT tissues accumulate significantly more lipid droplets compared to control mice (Fig. 5e, lower panels). Analysis of a subset of key metabolic genes including *Pparγ* revealed a drastic increase in their mRNA expression in the WAT (Fig. 5f). Leptin gene expression was also increased in Mmp11−/−WAT, while adiponectin expression was decreased. Consistent with the findings of increased FFA levels, we observed increased mRNA expression of the adipose triglyceride lipase (*Atgl*) lipolytic enzyme in the WAT of Mmp11−/−compared to WT mice (Fig. 5f). We also observed a significant increase in the expression of *Grb10* in the WAT of Mmp11−/−mice (Fig. 5f). Collectively, these results indicate enhanced adipogenesis in Mmp11−/−mice, which mirrors the Mmp11-Tg phenotype and strengthens the specific impact of MMP11 on adipose tissue metabolism. Consistent with decreased circulating IGF1 levels, the IGF1/AKT/FOXO1 signalling pathway was under-activated in the WAT of Mmp11−/−mice compared to WT animals (Fig. S4b).

Histological analysis of the livers of 12-week-old Mmp11−/−mice using oil red O staining revealed enhanced lipid droplet accumulation (Fig. S4c), and increased total triglyceride content (Fig. S4d), evoking hepatic steatosis. Consistently, we observed increased mRNA expression of *Pparγ*, fatty acid synthase (*Fas*), *Srebp1c* and *Chrebp* in the liver (Fig. S4e). We did not observe any changes in the expression of the β-oxidation gene *Pparα* (Fig. S4e). Moreover, increased expression of genes encoding the gluconeogenic enzymes glucose-6 phosphatase (G6Pase) and phosphoenolpyruvate carboxykinase (PCK1) was noted, together with a significant increase in the expression of *Foxo1*, a positive regulator of PCK1 and G6Pase in the liver^30^,^31^ (Fig. S4e). Consistent with a decrease in circulating IGF1 (Fig. 5d), we observed a concomitant increase in the expression of IGF-binding protein-1 (*Igfbp1*) in the livers of Mmp11−/−mice compared to controls (Fig. S4f). This supports the notion that MMP11 deficiency impacts on the insulin/IGF1-signalling pathway, through negative modulation of IGF1 bioavailability. Hence, a drastic decrease in AKT phosphorylation in the livers of MMP11-deficient animals compared to control mice was noted (Fig. S4g). Thus, MMP11 plays an important role in glucose and lipid metabolism as MMP11 ablation in mice induces diabesity with metabolic syndrome features and insulin resistance.

## Discussion

This study used gain- and loss-of-function *in vivo* models (Mmp11-Tg and Mmp11−/−mice, respectively) to uncover the role of MMP11 in whole body metabolism. We demonstrated that MMP11 plays a systemic role in the regulation of glucose and lipid homeostasis, which led to insulin sensitivity and protection from diabesity.

Although several studies have linked changes in MMP expression to obesity and insulin resistance, different MMPs have distinct activities. Likewise, insulin resistance and expanded adiposity induced by a sucrose-rich diet are associated with decreases in MMP2 and MMP9 in mouse adipose tissue^32^. *In vitro,* MMP2 promotes adipogenesis^33^, while MMP9 has no effect on adipocyte differentiation^34^. In human type 2 diabetes patients, MMP2 and MMP9 plasma levels are higher than those reported for healthy individuals^35^. MMP13 is linked to diet-induced obesity in mice and adipogenesis *in vitro*^36^. In addition, decreased MMP3 has been observed in the adipose tissue of retinoic acid receptor (RAR)-related orphan receptor gamma deficient (RORγ−/−) mice, which is associated with increased adipocyte formation and improved insulin sensitivity in diet-induced-obesity^37^. Furthermore, tissue inhibitors of MMPs (TIMPs) are also concerned. TIMP1 is increased in the serum and adipose tissue of obese mouse models, and TIMP1 injections in nutritionally-challenged mice lead to enlarged adipocytes^38^. Human type 2 diabetes patients exhibited increased plasma levels of TIMP1 and TIMP2^35^. On the other hand, TIMP3 deficiency in insulin receptor-haploinsufficient mice promotes type 2 diabetes and vascular inflammation^39^. Nonetheless, MMP11 appears to be unique in protecting against diabesity.

At the molecular level, we reported an approximate 20% increase in circulating IGF1 protein in Mmp11-Tg mice. Small variations in IGF1 have been shown to be significant *in vivo*. For example, a 7–8% increase in serum IGF1 constitutes a predictor of risk for prostate cancer^40^. Whether increased IGF1 bioavailability subsequent to MMP11 overexpression is a direct or indirect event will be difficult to decipher *in vivo*. Nevertheless, IGF1 levels are decreased in Mmp11−/−mice strongly supporting that MMP11 is responsible for IGF1 changes. Moreover, it is noteworthy that changes in IGF1 bioactivity are attributed to its release from IGF-binding proteins (IGFBPs), less than 5% of IGF1 being free in the plasma^41^,^42^. As IGFBP1 is one of the rare identified MMP11 substrates, MMP11 might regulate free IGF1 levels via IGFBP1 cleavage. Indeed, it has been shown *in vitro* that MMP11-dependent IGFBP1 cleavage leads to IGF1 release^5^.

Despite the positive effects of MMP11 on glucose homeostasis, MMP11 overexpression is linked to impaired mitochondrial function in the BAT due to increased oxidative stress and augmented expression of genes involved in aerobic glycolysis. Accordingly, additional *in vitro* experiments revealed that treatment of preadipocytes with recombinant MMP11 protein caused an alteration in mitochondrial function with a decrease in OXPHOS-mediated ATP production and enhanced proton leak. Mechanistically, this oxydative stress could be due to the MMP11-mediated increase in IGF1 bioavailability and consequent activation of IGF1/AKT/FOXO1 signalling in the adipose tissue. Supporting this hypothesis, activated AKT has been shown to promote intracellular ROS accumulation^43^ and induce aerobic glycolysis both in non-transformed cells^44^ and in cancer cells along with increased lactate production^45^. This MMP11-induced reduction in OXPHOS may provoke a substantial increase in systemic metabolism when energy demand is increased, thereby depleting adipose tissue lipid reserves. Furthermore, the browning of the WAT in Mmp11-Tg animals could be due to increased IGF1 signalling, as this pathway has been shown to positively regulate brown fat differentiation through two complementary pathways, RAS/ERK/CREB and PI3K/AKT/FOXO1^46^. To date, only one report links a metalloproteinase to mitochondrial dysfunction, as abnormalities in mitochondrial ultrastructure, impaired respiration and increased lipid peroxidation were observed during post-ischemic reperfusion in the heart of mice with cardiac specific overexpression of MMP2^47^.

Finally, our data highlight an unexpected important point. It is well established that aerobic glycolysis known as the “Warburg effect” is a hallmark of cancer cells to increase their biomass while decreasing OXPHOS^48^. Our data provide a rationale for the deleterious effects of MMP11 on human carcinomas. Given that MMP11 is overexpressed in almost all invasive cancers, we speculate that the effect of MMP11 overexpression observed in the adipose tissue of Mmp11-Tg model could recapitulate the metabolic changes observed in CAAs and CAFs^19^. This metabolic switch may provide cancer cells with metabolites such as fatty acids as we observed also increased BAT expression of the gene encoding the fatty acid oxidation enzyme long chain acyl Coenzyme A dehydrogenase or LCAD (data not shown) or through aerobic glycolysis (e.g. lactate). This energy transfer concept from CAFs to cancer cells is thought to occur after a ROS-induced catabolic response in CAFs that provides cancer cells with extranutrients to promote tumor progression^49^. Hence, due to the fact that MMP11 is expressed very early in tumor invasion processes, MMP11 could be one essential factor that initiates this metabolic reprogramming in CAAs and CAFs.

In conclusion, we provide *in vivo* evidence that MMP11 protects from diabesity (Fig. 5g) and promotes a metabolic switch from OXPHOS-induced ATP production to aerobic glycolysis to provide cells with complementary fuels to glucose (e.g. lactate, fatty acids) (Fig. 5h). We therefore propose that MMP11 be considered as an adipokine, which exerts an important systemic function under physiological conditions.

## Material and Methods

### Generation of mice

Mmp11−/−mice (on a 129 svJ genetic background) were previously described^12^. Mmp11-Tg mice express full-length mouse *Mmp11*. More details are provided in the supporting experimental procedure section. Mice were backcrossed 10 times onto the FVBN genetic background.

### Thermogenesis analyses

Body rectal temperature (BRT) was measured in Mmp11-Tg and control mice (n = 5/group) before placing the mice in a cold room (4 °C). BRT was then measured regularly at intervals of 1 h, up to 4 h. Mice were then placed in a normal temperature. For β3-adrenergic-induced thermogenesis, mice were injected intraperitoneally (i.p.) with BRL37344 (Sigma-Aldrich, France) and BRT was measured up to 3 h at intervals of 30 min.

### Histological analysis of liver and adipose tissue

Liver and adipose tissues (white: WAT and brown: BAT) were fixed immediately after sacrifice in paraformaldehyde 4% and then paraffin-embedded. 5 μm sections were stained with Hematoxylin & eosin (HE) and oil red O (for liver sections), and observed under a Leica™ light microscope.

### Transmission electron microscopy (TEM)

Tissues were fixed in 2.5% glutaraldehyde in 0.1 M sodium cacodylate buffer (pH 7.2) for 24 hr at 4 °C, washed in 0.1 M cacodylate buffer for 30 min and post-fixed in 1% osmium tetroxide in 0.1 M cacodylate buffer for 1 hr at 4 °C. Following stepwise dehydration with increasing concentrations of ethanol and embedding in Epon 812, ultrathin sections (70 nm) were stained with uranyl acetate and lead citrate, and observed with a Morgagni 268D electron microscope.

Mitochondrial function, western blot, enzymatic assays and RT-Q-PCR analyses methods are detailed in the supporting experimental procedures section associated to this manuscript.

This study has been approved by the IGBMC ethical committee for animal experimentation (Com’Eth). All methods were carried out in accordance with the approved guidelines

### Statistics

Results are expressed as mean ± standard error of mean (SEM). Statistical analyses were performed using a two-tailed Student’s t test for independent samples. Results were considered statistically significant when the p-value was <0.05. The degree of statistical significance was noted as: *p < 0.05, **p < 0.01, ***p < 0.001. All experiments were repeated independently at least 3 times.

## Additional Information

**How to cite this article**: Dali-Youcef, N. *et al.* Matrix metalloproteinase 11 protects from diabesity and promotes metabolic switch. *Sci. Rep.* **6**, 25140; doi: 10.1038/srep25140 (2016).

## Supplementary Material

Supplementary Information
