# Corrigendum: Matrix metalloproteinase 11 protects from diabesity and promotes metabolic switch

**DOI:** 10.1038/srep26916

**Published:** 2016-05-31

**Authors:** Nassim Dali-Youcef, Karim Hnia, Sébastien Blaise, Nadia Messaddeq, Stéphane Blanc, Catherine Postic, Philippe Valet, Catherine Tomasetto, Marie-Christine Rio

Scientific Reports 6: Article number: 2514010.1038/srep25140; published online: 04292016; updated: 05312016

In the PDF version of this Article, there is a typographical error in the Discussion section,

“Human type 2 diabetes patients exhibited increased plasma levels of TIMP1 and TIMP2^37^.”

should read:

“Human type 2 diabetes patients exhibited increased plasma levels of TIMP1 and TIMP2^35^.”

