# Supplementary material for: Matrix metalloproteinase 11 protects from diabesity and promotes metabolic switch
Source: Sci Rep. 2016 Apr 29;6:25140. doi: 10.1038/srep25140 (PMC4850390; doi:10.1038/srep25140)
Supplement: Supplementary Information [file srep25140-s1.doc]

**SUPPORTING INFORMATION**

Manuscript : **Matrix metalloproteinase 11 protects from diabesity and promotes metabolic switch**

By Nassim Dali-Youcef, Karim Hnia, Sébastien Blaise, Nadia Messaddeq, Stéphane Blanc, Catherine Postic, Philippe Valet, Catherine Tomasetto & Marie-Christine Rio

1. **Supplementary figures and legends:**

**
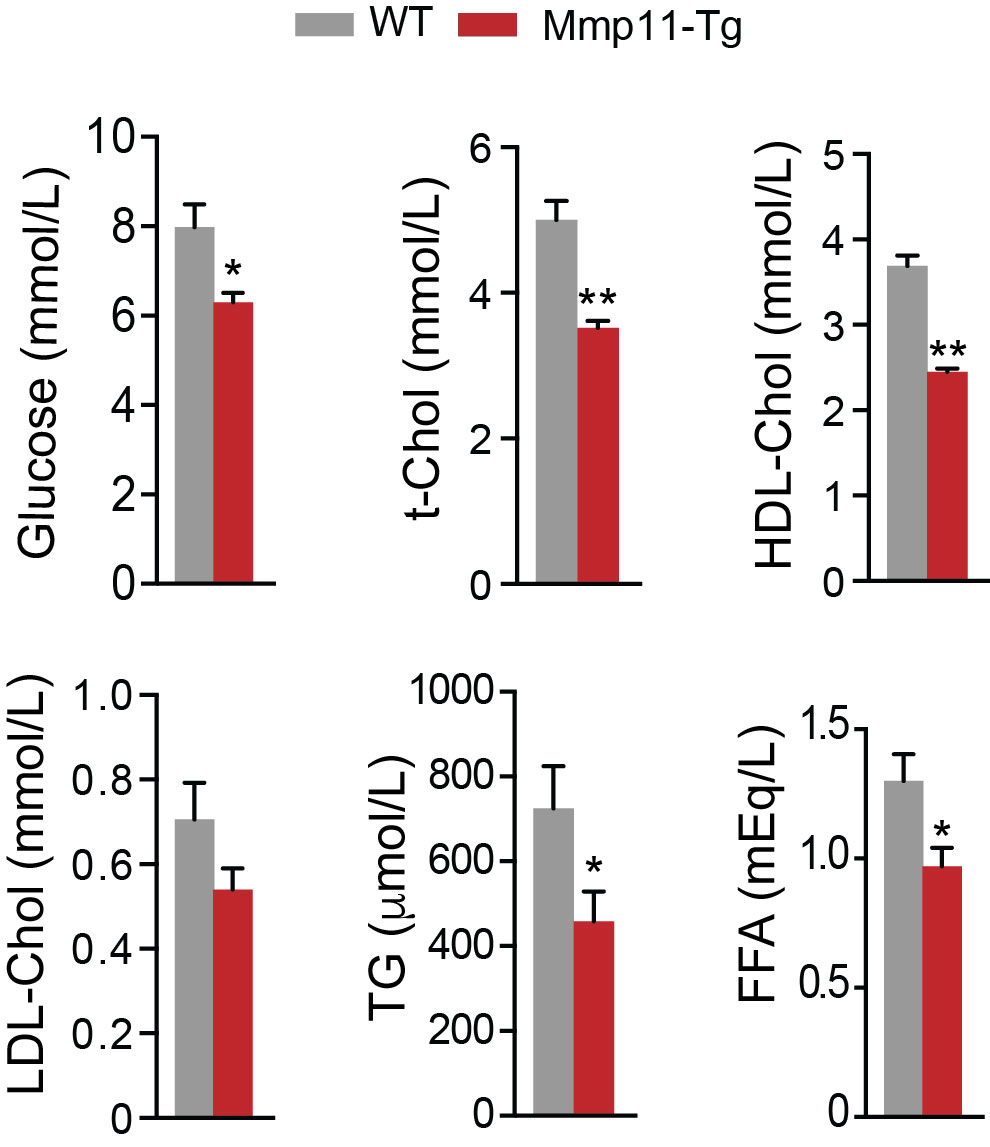
**

**Fig. S1 related to Fig. 1**

Circulating metabolic parameters in the serum of 12-week-old Mmp11-Tg and WT mice (n=7/group). Mmp11-Tg mice displayed decreased glucose, total cholesterol, HDL-cholesterol, triglycerides (TG) and free fatty acid (FFA).

**
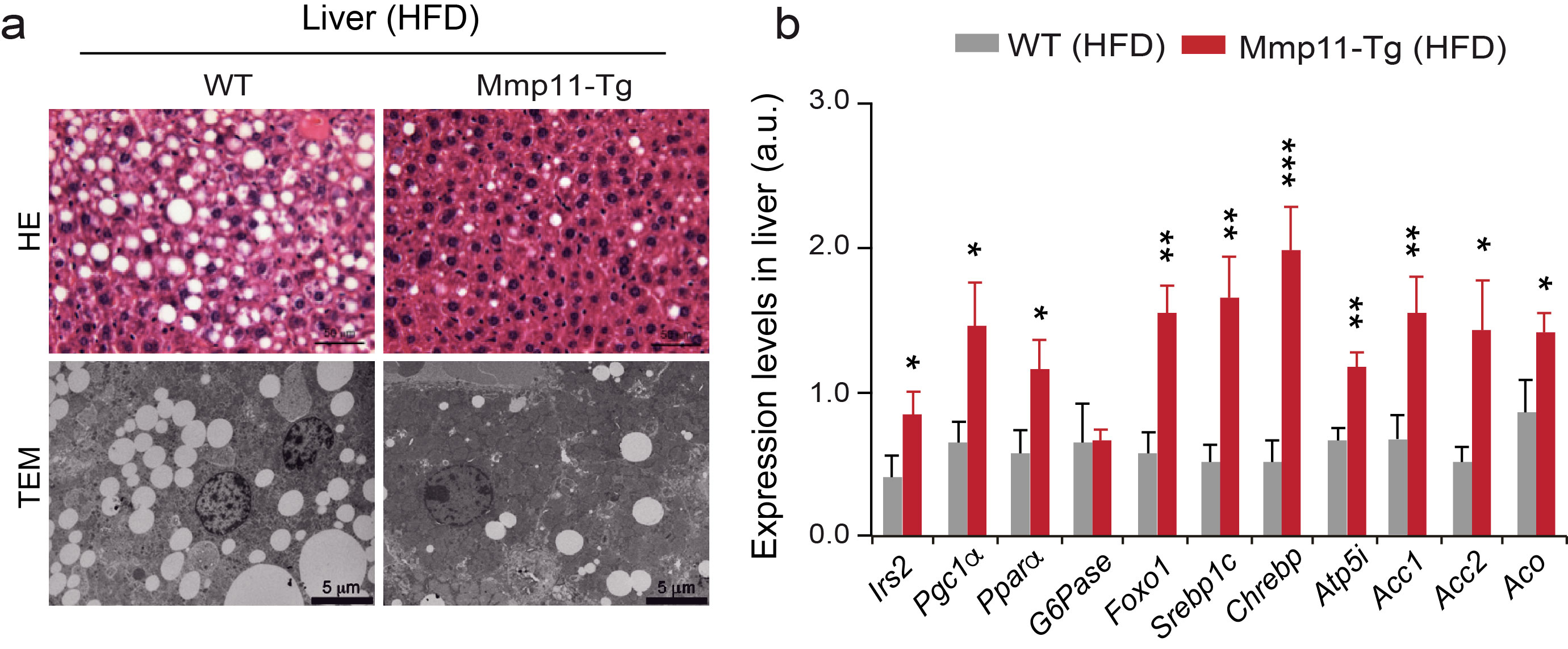
**

**Fig. S2 related to Fig. 3**

**a)** HE staining and TEM sections of liver from Mmp11-Tg and WT mice under HFD; **b)** Expression profiling of metabolic genes in Mmp11-Tg and WT mice under HFD (n=5-7/group).


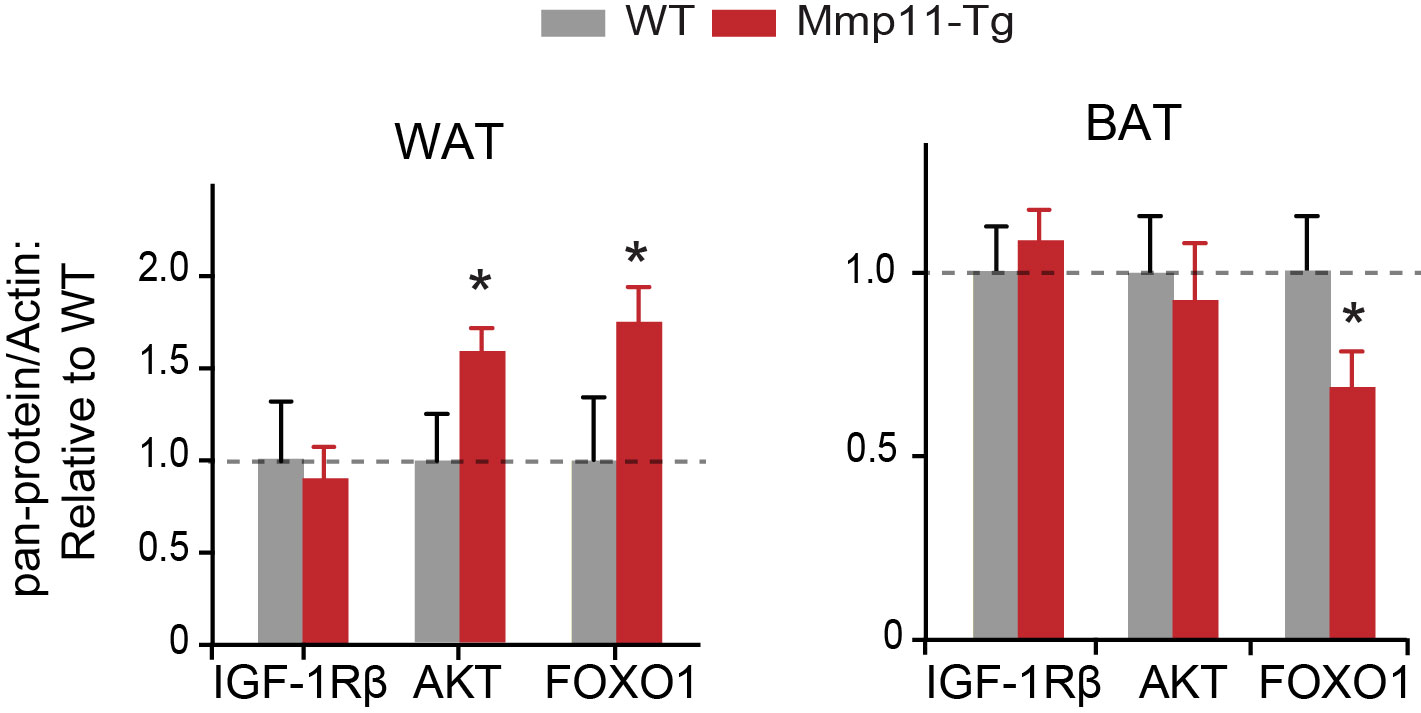


**Fig. S3 related to Fig. 4**

Relative WAT and BAT levels of IGF1Rβ, AKT and FOXO1 proteins from WT and Mmp11-Tg mice (n=6-8/group).

**
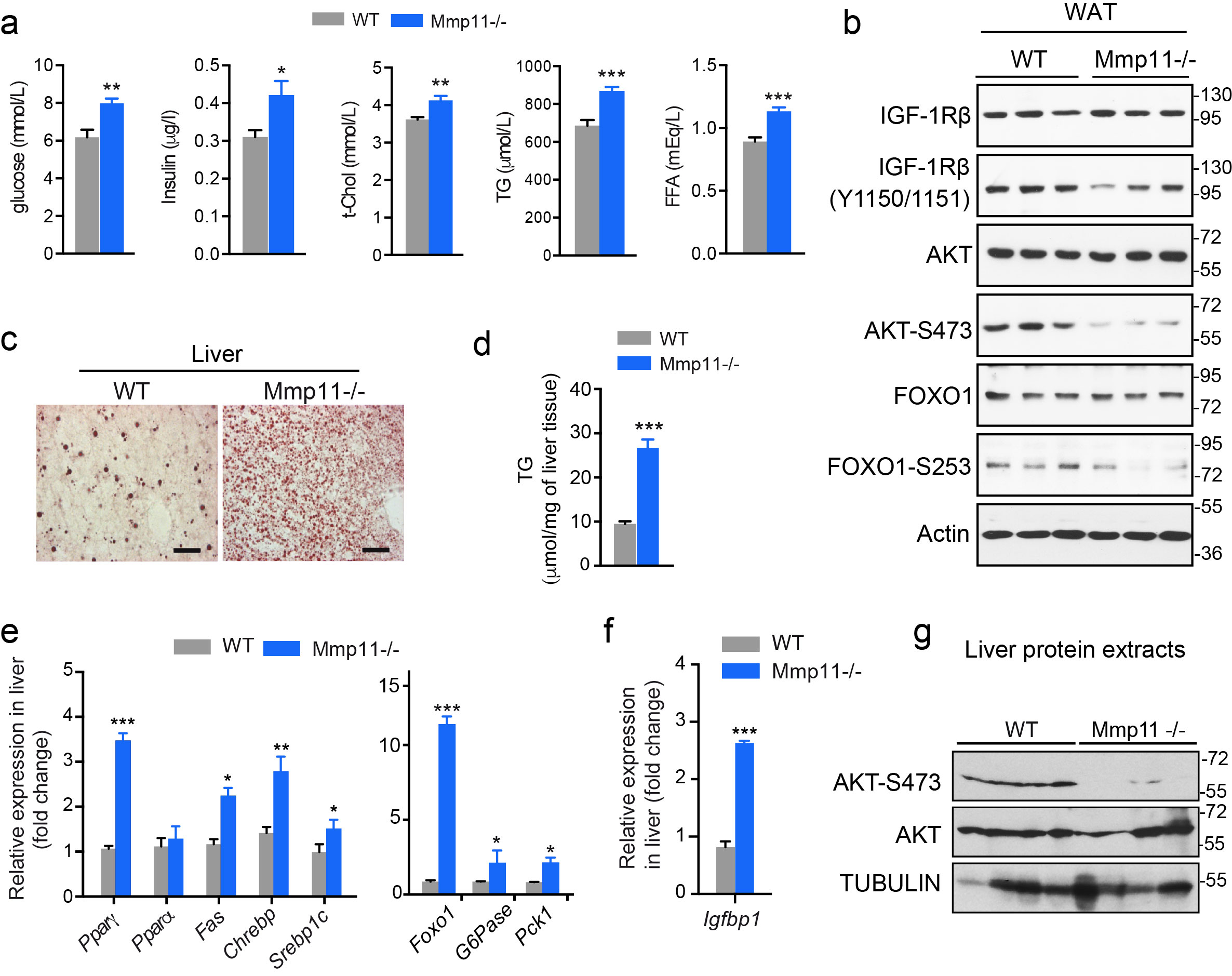
**

**Fig. S4 related to Fig. 5**

**a)** Biochemical parameters in the serum of 12-week aged Mmp11 -/- and WT mice. Mmp11 -/- displayed increased levels of glucose, insulin, t-cholesterol, TG and FFA compared to WT mice (n=8/group); **b)** Representative Immunoblots of IGF-1Rβ, pIGF-1Rβ (tyrosine 1150/1151), AKT, pAKT (serine 473), FOXO1 and pFOXO1 (serine 253) in WAT (n=5-6/group). **c)** Oil red-O staining of liver sections from Mmp11 -/- and WT mice; **d)** Total TG content in the liver of WT and Mmp11 -/- mice (n=5, 12-week-old); **e)** Expression profiling of lipogenic genes and of *Foxo1*, glucose-6 phosphatase (*G6pase*) and phosphoenolpyruvate carboxykinase (*Pck1*) in the liver of WT and Mmp11 -/- mice (n=7); **f)** *Igfbp1* transcripts in the liver of WT and Mmp11 -/- mice (n=7); **g)** AKT, S473-AKT and tubulin immunoblots in liver of WT and Mmp11 -/- mice.

**2) Supplementary experimental procedures**

***Generation of MMP11-Tg mice***

A cDNA encoding full-length mouse MMP11 (*mMmp11*) including the secretion signal was isolated by Sal I excision from a MMP11 pBluescript II vector. This *Mmp11* cDNA (1700 bp) was inserted in a pHR2 plasmid (a kind gift of S. Werner, Institute of Cell Biology, Zurich, Switzerland) ^1^, downstream of the human keratin 14 promoter (*K14*; 2000 bp) and rabbit beta-globin intron II (600 bp), and upstream of the regulatory elements of the human growth hormone gene poly(A) (*hGH* ; 600 bp). Standard procedures were followed in order to generate transgenic mice ^2^. Briefly, the *K14*/mMMP11/*hGH* poly A expression cassette (approximately 5000 bp) was excised from the plasmid to eliminate bacterial sequences, purified and microinjected into the pronucleus of FVBN mouse fertilized oocytes that were implanted into pseudopregnant females. Two founders were developed into lines and had a similar gross phenotype. The studies reported in this work were performed on a line with at least 20 *Mmp11* DNA copies. Heterozygous *K14-Mmp11* transgenic mice were further crossed with FVBN mice to obtain heterozygous *K14-Mmp11* and WT littermate mice. Offspring carrying the *K14-Mmp11* transgene were identified by PCR analysis of tail DNA using primers located at the border of exons 6 and 7 allowing the amplification of the *Mmp11* cDNA (transgene; product of 317 bp) or *Mmp11* genomic DNA (wild type; product of 1100 bp; including intron 6).

***Serum metabolic parameters***

Serum Glucose, insulin, total cholesterol (t-Chol), HDL-cholesterol (HDL-Chol), LDL-cholesterol (LDL-Chol), Triglycerides (TG), Free Fatty Acid (FFA), Adiponectin, Leptin and IGF1 were performed in the ICS metabolic platform (ICS-IGBMC, Illkirch, France) as previously described ^3^. IPGTT and IPIST analyses were performed according to the protocol as described ^4^.

***Western blot analyses***

Proteins were obtained after homogenization of frozen tissue in the following Buffer (50 mM Tris-Cl pH 7.5, 100 mM NaCl, 5 mM EDTA, 5 mM EGTA, 1 mM DTT, 0,5% Triton X-100, 2 mM PMSF) supplemented with complete protease inhibitor tablet (Roche, France) and 1mM Leupeptin and 1mM pepstatin A (Sigma, France) to avoid protein degradation. Protein homogenates were mixed with 4x Laemmli buffer, boiled 5 min at 95°C, separated on 10 or 12 % SDS polyacrylamide gels and transferred onto nitrocellulose membranes. After blocking the membranes overnight at 4 °C in 5% milk in TBST, primary antibodies were applied at the specific dilution in 5% milk in TBST buffer and incubated for 1 hr at RT or overnight at 4°C depending on the specificity of the antibodies and manufacturer's instructions. After adding the primary antibody, the membranes were washed 5 times with TBST, and horseradish peroxidase-conjugated secondary antibodies (Jackson Immuno Research Laboratories, UK) were applied for 1 hr. After 5 times washing with TBST, membranes were developed using superSignal west Pico kit (Thermo scientific, PEIRCE) or revealed in the Amersham™ Imager 600 (GE Helthcare, Life sciences). The antibodies used for western blot were, the rabbit polyclonal anti- AKT, p-AKT (S256), FOXO1, p-FOXO1 (S253), IGF1, IGF1Rβ and phospho IGF1-Rβ (Y1150/1151) from cell signaling, rabbit polyclonal anti-MMP11 N°3143 (IGBMC, Illkirch, France), anti-tubulin N°12G10 (Developmental Studies Hybridoma Bank, IO, USA), and anti-actin N°2D7 (IGBMC, Illkirch, France). Rabbit polyclonal anti-Glutathione Peroxidase 1 and anti-Catalase were purchased from Abcam (UK).

***Gene expression analyses by real time quantitative RT-PCR***

Frozen specimen (-80°C) were homogenized in either QIAzol® (Qiagen, Courtaboeuf, France) for lipid rich tissues (adipose tissues) or TRIzol® (ambion by life technologies, Paisley, UK) for liver, and total RNA was extracted following the manufacturer’s instruction as previously described ^5^. One μg of total RNA were reverse transcribed at 42°C for 50 min using SuperScript™ II reverse transcriptase (200 U/μL, Invitrogen by Life technologies, Paisley, UK) following the manufacturer’s protocol. Gene expression levels were quantified by real-time quantitative RT-PCR using [SYBR® Green JumpStart™ Taq ReadyMix™](http://www.sigmaaldrich.com/catalog/product/sigma/s4438) (Sigma-Aldrich, St-Quentin Fallavier, France) and specific primers listed in Table S1.

***Mitochondrial function and membrane potential***

Recombinant active- and inactive-MMP11proteins were added to the culture medium for 72 h at a concentration of 3.5 μg/ml and the medium was changed every 24 h, as recombinant MMP11 is susceptible to autolysis ^6^. All mitochondrial parameters were calculated according to XF Cell Mito Stress test Kit (Seahorse Bioscience) and as previously described ^7^. Mitochondrial function was performed using the XF Cell Mito Stress test Kit (Seahorse Bioscience) according to manufacture’s instructions. Pre-adipocyte 3T3-L1 cells were treated with Vehicle (50 mM Tris-Hcl, EDTA buffer PH 8) or MMP11 catalytic domain (active) or the inactive MMP11 as described previously ^8^. For mitochondrial membrane potential, 3T3-L1 treated cells were stained with 10 nM Tetramethylrhodamine, methyl ester (TMRM, Molecular Probes, Life Technologies Inc.) for 30 minutes. Fluorescence of the cells was quantified in basal conditions (time 0) by live fluorescence microscopy (excitation/emission fluorescence ratio 549 nm/ 573 nm), using an inverted fluorescent microscope. Background fluorescence was measured and subtracted from the total fluorescence intensity. Images were analysed using ImageJ analysis software (NIH).

SRC is defined as the difference between the FCCP-induced maximal respiration and the basal respiration. Proton leak is a measure of the remaining respiration not coupled to ATP production.

***Lipid peroxidation Catalase and glutathione peroxidase (GPx) enzymatic assays***

Lipid peroxidation (TBARS) were performed by evaluating the Malondialdehyde (MDA) levels in BAT homogenates containing 250 μl of RIPA Buffer with protease inhibitors (PMSF and EDTA free Roche tablet) and then Sonicated for 15 seconds at 40V on ice. The supernatant was used for the dosage after centrifugation at 1,600 x g for 10 minutes at 4°C. The TBARS assay was then performed according to the manufacturer's protocol (Cayman Chemical Company, Ann Arbor, MI), and the reaction products were measured spectrophotometrically at 532 nm and normalized to a standard curve.

Tissue GPx enzyme activity was measured using the Glutathione Peroxidase Assay Kit (Colorimetric) (ab102530) according to the manufacturer recommendations. GPx converts reduced glutathione (GSH) to oxidized glutathione (GSSG) while reducing lipid hydroperoxides to their corresponding alcohols or free hydrogen peroxide to water. The generated GSSG is reduced to GSH with consumption of NADPH by GR. The decrease of NADPH (measured at 340 nm) is proportional to GPx activity, which was calculated in Unit/mg protein. One unit is defined as the amount of enzyme that will cause the oxidation of 1.0 μmol of NADPH to NADP+ under the assay kit condition per minute at 25°C.

Catalase activity was measured by catalase Assay Kit (colorimetric/fluorometric) (ab83464) according to manufacture recommendation. Catalase first reacts with H_2_O_2_ to produce water and oxygen, the unconverted H_2_O_2_ reacts with OxiRed probe to produce a product, which can be measured at 570 nm (Colorimetric method) or at Ex/Em = 535/587 nm (fluorometric method). Catalase activity is reversely proportional to the signal. One unit of catalase is the amount of catalase that decomposes 1.0 μmol of H_2_O_2_ per minute at pH 4.5 at 25°C.

***Total energy expenditure (TEE) measurement***

Daily energy expenditure (DEE) was determined during a 2-day period by the two-point doubly labeled water method (DLW) ^9^. After baseline blood sampling, a premixed 2 g/(kg estimated total body water, TBW) dose of DLW was intravenously injected to the animals. The dose was composed of 0.55 g/(kg estimated TBW) 97% H_2_^18^O (Rotem Industries Ltd., Israel) and 0.15 g/(kg estimated TBW) 99.9% ^2^H_2_O (Cambridge Isotope Laboratories, Andover, MA, USA) and was diluted with 3% NaCl to physiological osmolarity. We assumed a percentage of hydration of 0.60 to calculate doses. The doses were calculated to ensure an in vivo enrichment of about 250 and 1200‰ for 18-oxygen and deuterium, respectively. Isotopic equilibration in body water was determined from a blood sample collected at 1-h post-dose. Fluxes were calculated by the use of a last blood sample 2-days post-dose. Immediately after collection, blood-containing capillaries were rapidly flame-sealed.

Water from serum and urine samples was extracted by cryo-distilation. 0.1 µL of water was reduced to hydrogen and carbon monoxide by reduction on a glassy carbon reactor held at 1400°C in an elemental analyzer (Flash HT; ThermoFisher Germany). Hydrogen and carbon monoxide gases were separated by a GC column held at 104°C coupled to a continuous-flow Delta-V isotope ratio mass spectrometer. Isotopic abundances of deuterium and 18-oxygen in hydrogen and carbon monoxide gazes were measured in quintuplicate and repeated if SD exceeded 2 and 0.5‰, respectively. All enrichments were expressed against International Atomic Energy Agency standards.

CO_2_ production was calculated according to the single pool equation of Speakman, validated in Blanc et al. ^10^. DEE was calculated by the Weir's equation as described ^10^. Total body water (TBW) was measured from the dilution space of 18-oxygen after correction for exchange by the factor 1.007. Free fat mass (FFM) was calculated from TBW by assuming hydration coefficient of 73.2%. Fat mass (FM) was calculated by the difference of FFM from the body mass.

***Supplementary experimental procedures references***

1 Munz, B. *et al.* Overexpression of activin A in the skin of transgenic mice reveals new activities of activin in epidermal morphogenesis, dermal fibrosis and wound repair. *Embo J* **18**, 5205-5215, doi:10.1093/emboj/18.19.5205 (1999).

2 Tomasetto, C. *et al.* Breast cancer protein PS2 synthesis in mammary gland of transgenic mice and secretion into milk. *Mol Endocrinol* **3**, 1579-1584, doi:10.1210/mend-3-10-1579 (1989).

3 Champy, M. F. *et al.* Genetic background determines metabolic phenotypes in the mouse. *Mamm Genome* **19**, 318-331, doi:10.1007/s00335-008-9107-z (2008).

4 Heikkinen, S., Argmann, C. A., Champy, M. F. & Auwerx, J. Evaluation of glucose homeostasis. *Curr Protoc Mol Biol* **Chapter 29**, Unit 29B 23, doi:10.1002/0471142727.mb29b03s77 (2007).

5 Chomczynski, P. A reagent for the single-step simultaneous isolation of RNA, DNA and proteins from cell and tissue samples. *Biotechniques* **15**, 532-534, 536-537 (1993).

6 Noel, A. *et al.* Identification of structural determinants controlling human and mouse stromelysin-3 proteolytic activities. *J Biol Chem* **270**, 22866-22872 (1995).

7 Gavriilidis, C., Miwa, S., von Zglinicki, T., Taylor, R. W. & Young, D. A. Mitochondrial dysfunction in osteoarthritis is associated with down-regulation of superoxide dismutase 2. *Arthritis and rheumatism* **65**, 378-387, doi:10.1002/art.37782 (2013).

8 Andarawewa, K. L. *et al.* Stromelysin-3 is a potent negative regulator of adipogenesis participating to cancer cell-adipocyte interaction/crosstalk at the tumor invasive front. *Cancer Res* **65**, 10862-10871, doi:10.1158/0008-5472.CAN-05-1231 (2005).

9 Schoeller, D. A. *et al.* Energy expenditure by doubly labeled water: validation in humans and proposed calculation. *Am J Physiol* **250**, R823-830 (1986).

10 Blanc, S., Geloen, A., Pachiaudi, C., Gharib, C. & Normand, S. Validation of the doubly labeled water method in rats during isolation and simulated weightlessness. *Am J Physiol Regul Integr Comp Physiol* **279**, R1964-1979 (2000).

1. **Table S1**. Primers used for real-time quantitative-PCR

|  | Forward | Reverse |
| --- | --- | --- |
| *18s* | GGGAGCCTGAGAAACGGC | GGGTCGGGAGTGGGTAATTTT |
| *Pgc1α* | ACAGCTTTCTGGGTGGATTG | AGGGCAATCCGTCTTCATC |
| *Pparγ* | TGGCCACCTCTTTGCTCTGCTC | AGGCCGAGAAGGAGAAGCTGTTG |
| *Fas* | ACCCAAGCATCATTTTCGTC | AGGATATGGAGAGGGCTGGT |
| *Leptin* | GACACCAAAACCCTCAT | CAGTGTCTGGTCCATCT |
| *Adipoq* | GCCAGTCATGCCGAAGA | TCTCCAGCCCCACACTGAAC |
| *Resistin* | TTCCTTGTCCCTGAACTGCTG | GCTGGAAACCACGCTCACTT |
| *Atgl* | GACCTGATGACCACCCTTTC | CAGATACTGGCAGATGCTACC |
| *Igfbp1* | GGAGATCGCCGACCTCAAG | CTGCAGCTAATCTCTCTCTAGCACT |
| *Glut4* | CTTCTTTGAGATTGGCCCTGG | AGGTGAAGATGAAGAAGCCAAG |
| *Foxo1* | AAGAGCGTGCCCTACTTCAA | CTCCCTCTGGATTGAGCATC |
| *Srebp1c* | AACGTCACTTCCAGCTAGAC | CCACTAAGGTGCCTACAGAGC |
| *Chrebp* | CTGGGGACCTAAACAGGAGC | GAAGCCACCCTATAGCTCCC |
| *Pck1* | CCACAGCTGCTGCAGAACA | GAAGGGTCGCATGGCAAA |
| *G6Pase* | CCGGATCTACCTTGCTGCTCAC | TAGCAGGTAGAATCCAAGCGCG |
| *Acc1* | GACAGACTGATCGCAGAGAAAG | TGGAGAGCCCCACACACA |
| *Acc2* | CCCAGCCGAGTTTGTCACT | GGCGATGAGCACCTTCTCTA |
| *Aco* | CCCAACTGTGACTTCCATT | GGCATGTAACCCGTAGCACT |
| *Pparα* | AGGAAGCCGTTCTGTGACAT | TTGAAGGAGCTTTGGGAAGA |
| *Tnfα* | TGGGACAGTGACCTGGACTGT | TTCGGAAAGCCCATTTGAGT |
| *Bmp4* | TGAGGAGTTTCCATCACGAA | TTATACGGTGGAAGCCCTGT |
| *Bmp7* | AGCGATTTGACAACGAGACC | AGCTGTAAGCCCAGGTTGTG |
| *Cidea* | CTCGGCTGTCTCAATGTCAA | CCGCATAGACCAGGAACTGT |
| *Ucp1* | CCGAAACTGTACAGCGGTCT | TAAGCCGGCTGAGATCTTGT |
| *Ldha* | AGACAAACTCAAGGGCGAGA | CAGCTTGCAGTGTGGACTGT |
| *Ldhb* | TAAGCACCGTGTGATTGGAA | AGACTCCTGCCACATTCACC |
| *Mct1* | GCATTTCCCAAATCCATCAC | CGGCTGCCGTATTTATTCAC |
| *Pdh* | TCGAAGCCATAGAAGCCAGT | AGGCATAGGGACATCAGCAC |
| *Dld* | TGGCAGCACTCAGGTTATTG | ACTGAACCCAGTTCCACACC |
| *Dlat* | TCCTGCAGGTGTCTTCACAG | GACGGAGATTTTCCCTTTCC |
| *Atp5i* | CCCCTGCTGAAATCCCTACA | TAAAACCACATCCACACCTC |
| *Irs1* | GCGGGCTGACTCCAAGAAC | GCTATCCGCGGCAATGG |
| *Irs2* | GGAGAACCCAGACCCTAAGCTAC | GATGCCTTTGAGGCCTTCAC |
| *Grb10* | AAGAAGCAGTACAACGCGCC | CTCATCTTCGGCACAGAGCA |
| *C/ebpb* | tgatgcaatccggatcaa | cacgtgtgttgcgtcagtc |
| *Prdm16* | acaggcaggctaagaaccag | cgtggagaggagtgtcttcag |
| *Tfam* | agggagctaccagaagcaga | cccatcagctgacttggagt |
| *CD137* | gctggccctgatcttcatta | agctgctccagtggtcttct |
| *Cited1* | gaggcctgcacttgatgtc | tggagtaggccagagagttca |
